# Sharing the World—A Social Aspect of Consciousness

**DOI:** 10.1162/opmi.a.5

**Published:** 2025-06-25

**Authors:** Chris Frith

**Affiliations:** Institute of Philosophy, School of Advanced Study, University of London, London, UK; Functional Imaging Laboratory, University College London, London, UK

**Keywords:** cognition, consciousness, corollary discharge, allocentric representation, common knowledge, coordination, cooperation

## Abstract

Moving through our environment generates multiple changes in my sensations. But I do not experience the environment as changing. My conscious perceptual experience is of a stable environment through which I move. This perception is created by intricate neural computations that automatically take account of my movements. The stable environment that I experience is independent of my actions. As a result, I experience it as objective: a set of facts about the world that constrain my movements. Because it is objective I expect that it will also constrain the movements of others in the same way, whether these are rocks rolling down a hill or animals foraging for food. This experience of objectivity creates a shared understanding of the world that enhances our interactions with others. Our perceptual experiences, while personal, are shaped by our model of the world, and since others are modelling the same world, their models will be very similar. Interactions with others will further increase this similarity. The models create a form of common knowledge. This common knowledge is an inherent feature of our basic conscious perception, even when we’re not actively reflecting on or deliberately sharing our experiences. The common knowledge created by our conscious perception of the world enables the coordination of behaviour which is a critical precursor for the evolution of cooperative behaviour.

## INTRODUCTION

Being conscious—having subjective experiences—is a process that has *evolved*, eventually emerging in creatures with sufficiently complex nervous systems (Bronfman et al., 2016; Carruthers, 2000). But what selective advantages did consciousness confer that drove this evolution? One suggestion is that the development of consciousness occurred in parallel with increases in intelligent behaviour, particularly the ability to solve novel problems (e.g., Seed & Mayer, 2017). In a version of this idea, Humphrey (1987) suggested that consciousness emerged in response to the novel problems associated with the complexities of human social life. A form of advanced social intelligence was needed so that people could model the minds of others (see also Frith, 1995). Carruthers (2009) proposed that consciousness emerged when this capacity to read the minds of others was applied to the self.

These suggestions clearly relate to a reflective form of consciousness, also known as meta-consciousness (Schooler, 2002) or C2 (Dehaene et al., 2017), which is probably unique to humans. This process of reflection enables us to talk about our experiences and so explicitly share them with other people. The ability to talk about our subjective experience confers many advantages. By sharing feelings, such as our degree of confidence in a choice, we can make group decisions that are better than individual decisions (Bahrami et al., 2010). In the longer term, sharing experiences enables us to interpret our feelings better and develop increasingly accurate, shared models of the world and our place in it (Heyes et al., 2020). As a result, our ability to reflect on our subjective experiences has a critical role in the development of cumulative culture (Birch & Heyes, 2021; Frith, 2023).

However, most of the time, our subjective experience, in particular our perceptual experience of the physical world around us occurs in the absence of reflection. This is a non-reflective aspect of consciousness which is likely to be present in many animals (e.g., Birch et al., 2020). This form of consciousness is essentially private, since, in the absence of reflection, it cannot be deliberately shared with others. Here I will argue that even in the absence of any explicit intention to share, this non-reflective form of consciousness still has an aspect of sharing. This sharing is a form of common knowledge that is involuntary and indiscriminate. But why might it be important for evolution? I will start by considering a problem that is faced by all those animal species that move through the world under their own power.

## HOW IS IT THAT WE EXPERIENCE THE WORLD AS STATIONARY WHEN WE MOVE THROUGH IT?

When animals evolved the ability to move through the world under their own power, they also developed complex neural mechanisms to interpret the information arising from the senses. Every movement generates sensory signals (*re-afference*). If we took these signals at face value, the visual world, for example, would seem to be continuously jumping about. And this would happen even when we were standing still, since our eyes are continuously moving. Nevertheless, we have the vivid subjective experience of moving through a fixed and stationary world. How is this stable model of the world created from such variable and volatile inputs?

**Figure 1.**
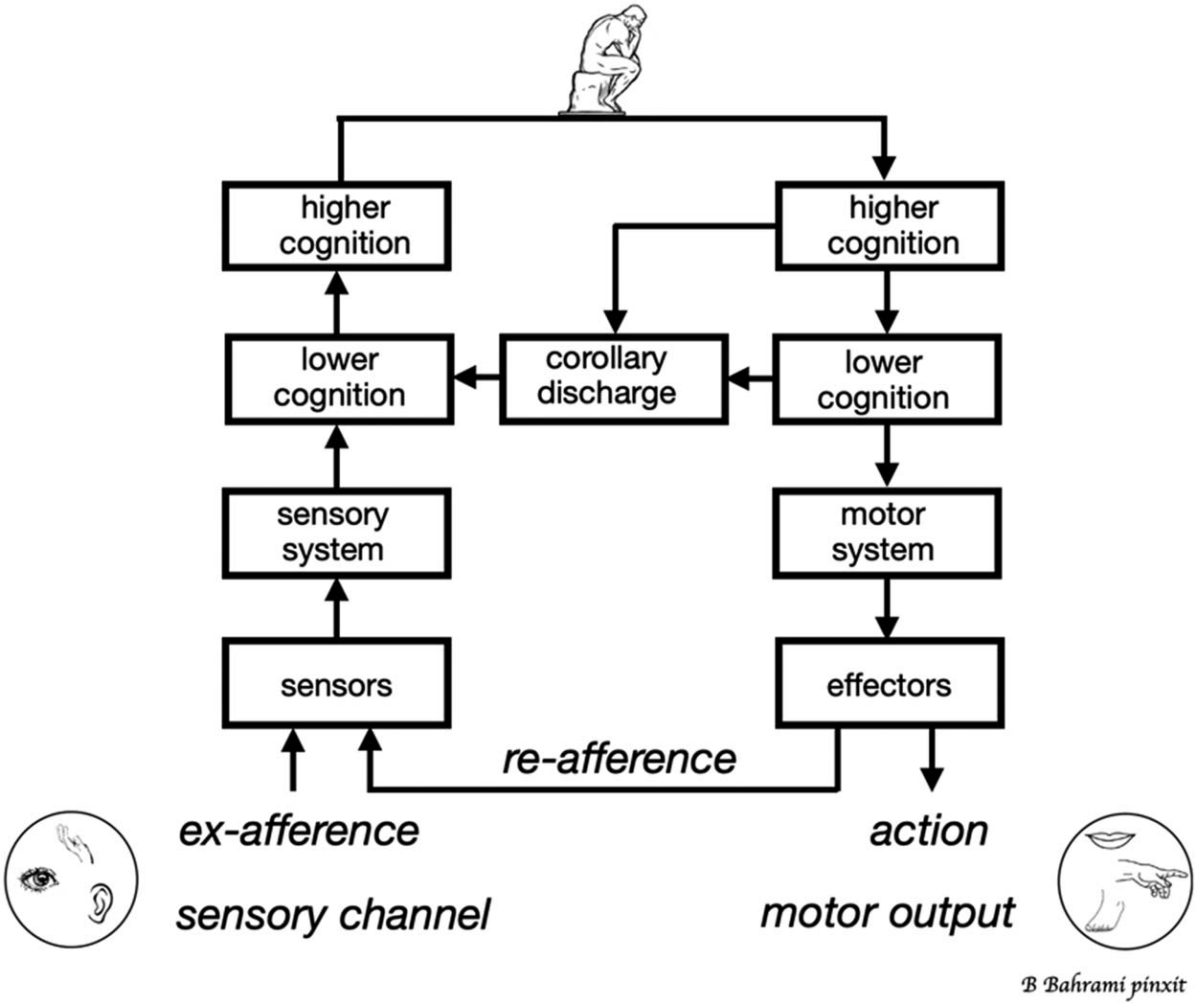
Corollary discharge: A mechanism for creating stability (after Crapse & Sommer, 2008). Actions from the motor system cause inputs (re-afference) to the sensory system. Corollary discharge signals from the motor system enable the sensory system to take account of these re-afferent signals. At a lower level of cognition, corollary discharge signals can be used to suppress inappropriate reflexive responses to re-afferent inputs. At a higher level of cognition, the corollary discharge signals can be used to create a stable representation of the environment.

The problem is solved, in part, by the corollary discharge mechanism. Corollary discharge is a neural signal that originates in the same sensory motor region that generates the command to make a movement (see Figure 1). This signal is sent to the brain regions that need to take account of the forthcoming movement. In animal species with nervous systems the corollary discharge mechanism can compensate for sensory changes by registering the associated action (Jékely et al., 2021). Humphrey (2006; see also Vallortigara, 2021) suggested that corollary discharge might have a critical role in creating phenomenal consciousness. The idea is that the corollary discharge signal enables us to make a distinction between what is experienced as happening out there (perception) and what is experienced as happening to me (feeling). Here, I shall concentrate on perception, the representation of what’s out there.

While corollary discharge is not sufficient on its own to generate the representation of the stable world that underlies conscious experience, it is a crucial prerequisite. Take *C. elegans*, a tiny worm that can move backwards and forwards in response to environmental signals. The problem it must solve is how to sustain forward movement, despite fluctuations in sensory input. Normally, when the front of the worm is stimulated, backward movement is initiated to avoid obstacles or potential dangers. But such stimulation also occurs when the worm attempts to move forward. Corollary discharge signals enable a distinction to be made between externally generated (*ex-afference*) and self-generated stimulation (*re-afference*). This enables the reflexive response of moving backwards when the front is stimulated to be suppressed so that the creature can continue to move forward (Ji et al., 2021) (lower cognition in Figure 1).

In this example, corollary discharge signals are sufficient to sustain goal-directed movement. But they are probably not sufficient to create a representation of a stable environment. For this a more complex nervous system is needed. Many species with more complex nervous systems, including fish, bats, and rats, can use corollary discharge to construct stable internal representations of the environment. In monkeys, for example, a more complex computation is used to transform an unstable retinotopic map into a stable representation (Cavanaugh et al., 2016) (higher cognition in Figure 1). Here, corollary discharge signals associated with eye movements are used to create stable visual perception. I suggest that this mental representation of a stable environment through which we move is a key component of our subjective experience of an objective reality.

This stable representation of environmental space is known as a cognitive map (Tolman, 1948). It enhances sensory motor learning and planning (Crapse & Sommer, 2008) and also enables us to simulate the possible effects of our behaviour (Craik, 1967). This map is an *allocentric* representation. In such a representation the environment is represented in terms of the relations between objects in the external world and is independent of the current location of the viewer (Dong et al., 2024). This contrasts with an *egocentric* representation which is anchored at our current location in the environment and changes as we move. This egocentric representation is critical for actions such as reaching and grasping.

Is there any evidence linking allocentric representations to consciousness? There is, indeed, evidence that actions are generally more egocentric while conscious perceptual experience is more allocentric (see McBeath et al., 2018 for a review). This distinction is particularly striking in the case of DF, a patient who acquired visual agnosia after damage to the lateral occipital cortex. Her behaviour provides strong evidence of such a link. DF is not conventionally blind but is not aware of the shapes of objects. So, she cannot recognise what they are, name them, or report how they are oriented. But, when she reaches and grasps objects, she grasps them correctly. Her grasp automatically takes account of their shape (Goodale et al., 1991). This ability is sustained by ever changing egocentric representations, which guide her hand to the object. This process appears to be intact. However, visual information about the shape of the objects that she is grasping correctly is only available for control of her movements. It does not enter her subjective experience. This result might superficially seem to show that DF is impaired on perceptual tasks, but not on motor tasks. However, Schenk (2006) showed that DF’s performance is preserved in both perceptual and visuomotor tasks when the required spatial information is hand-centred (*which dot is nearer to your finger*, egocentric) but impaired when the information is object-centred (*which dot is nearer to the cross*, allocentric). It seems that, in the absence of an allocentric representation, DF can have no conscious experience of visual shape.

## EXPERIENCING AN OBJECTIVE WORLD

Following this argument, the allocentric representation allows me to have the experience of moving through a stationary world. However, I don’t just experience the spatial environment as stationary as I move through it. I experience it as an objective reality. How is this experience created?

At the sub-personal level many complex computations, starting with the analysis of corollary discharge signals, are needed to achieve a representation of a stable environment (Grush, 2000), but, at the personal level, we remain unaware of all these processes, and ‘see through them’. We experience ourselves as having direct contact with our environment. This transparency of perception is a key feature of consciousness (Metzinger, 2003). We don’t experience our environment as a constructed representation. We experience ourselves as active agents within an objective, spatial realm that exists independently of us. Grush (2000) articulates this as follows “That I am a subject of experience in an independent world is made possible by my interpreting my experience as being from a particular point of view within an allocentric spatial realm that contains and is independent of that point of view” (see also Strawson, 1959). In other words, I have the subjective experience of an objective (allocentric) world from my own (egocentric) viewpoint.

The allocentric nature of this representation helps us to feel that we are in contact with an objective world. I would go further and suggest that we have a strong belief in an objective world that exists independently of us. We believe that it remains even when we are no longer there. As Hume said, *“…, we always suppose an external universe, which depends not on our perception, but would exist, though we and every sensible creature were absent or annihilated. Even the animal creation are governed by a like opinion …”* (Hume, 1748, Section XII, part I). I experience it as an objective reality: a set of facts about the world that constrain my movements. I believe it stays the same even though I experience it from different points of view.

This feeling of objectivity is sometimes called *presence* (Seth, 2014). The experience of *presence* is key to the success of virtual reality systems. Presence is “*the user’s feeling of being physically in the virtual environment and the ability to interact with that environment and the objects in it as if it were real”* (Caroux, 2023). It is the feeling of presence that puts the reality into virtual reality, but this feeling of objectivity is difficult to create. The displays in virtual reality must provide precisely the same sensory information to an observer, contingent upon the observers’ movements, as the observer would receive if he/she were actually present in the simulated environment (Loomis, 1992). This requires reverse engineering of the mechanisms by which the brain creates a stable model of the environment and is yet to be fully achieved by VR systems. In contrast, in the real world, however, as opposed to virtual worlds, our experience of that world as reality is almost unshakeable.

What is the basis of this unshakeable feeling? My experience is based on my allocentric model of the spatial environment. But the experience only achieves its stability and its presence because my sensations feel exactly as predicted, when I move^1^. It is this sufficiently exact predictability that is yet to be achieved by VR technology. So, to achieve the experience of objectivity, in addition to my model of the environment, I need an estimate of the accuracy and precision of this model.

## OBJECTIVE REALITY NEEDS HIGH PRECISION

In a recent variant of higher order thought theories of consciousness, Hakwan Lau proposed that, for the conviction that a subjective experience is an ‘objective reality’ (rather than being a dream or a vivid memory), our first-order representation of the environment must be certified as ‘real’ by a higher-order state (perceptual reality monitoring; Lau, 2022). This certification comes in the form of an estimate of the accuracy and precision of our model.

Lau suggests that the neural mechanism for this process may resemble that of Generative Adversarial Networks (GANs; Gershman, 2019). These networks have been remarkably successful in generating realistic, but non-existent faces (Tucciarelli et al., 2022). One part of the network, the generator, learns to create realistic looking faces. The other part, the discriminator, learns to detect ‘fake’ faces. The competition between these two parts speeds up and enhances the learning process. When the generator has learned to fool the discriminator with ‘fake’ faces, then these have become sufficiently accurate representations of real faces. This could be the computational mechanism in the brain through which representations of our environment become sufficiently accurate and precise to acquire a certification of objectivity and achieve presence.

Lau gives the example of an infant looking at a rabbit. In this example, a discriminator, probably instantiated in the prefrontal cortex, indicates that the precision of the model of a rabbit, inferred from sensory inputs and represented in higher order visual cortex, is sufficient to be a true reflection of the world (see Brown et al., 2019). There really is a rabbit out there. According to Lau, it is this certification process that gives the assertoric force necessary for subjective experience. Nevertheless, however strong the assertoric force, it is still possible that this perception might be a hallucination.

## AN OBJECTIVE WORLD CONSTRAINS THE BEHAVIOUR OF OTHERS

The objectivity of my model, a set of facts about the world, will constrain the behaviour of others just as it constrains my own behaviour. An obstacle that constrains how I can move will also constrain how others will move.

**Figure 2.**
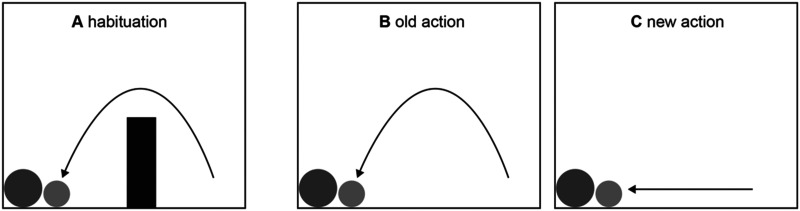
An infant repeatedly sees the small ball jumping over an obstacle to reach the large ball (A). Then the obstacle is removed, and the small ball continues to jump (B) or moves directly to the large ball (C). The infant is surprised if the small ball continues to jump (Redrawn from Figure 1 in Csibra et al., 1999.).

Expectations about how agents will move through the world can already be observed in human infants. After seeing an agent repeatedly jump over an obstacle to reach its goal, a 9-month-old infant expects the agent to move straight to the goal when the obstacle is removed (e.g., Csibra et al., 1999; see Figure 2).

This observation supports the idea that infants expect agents to behave rationally, minimizing the cost of their action by taking the most efficient route to the goal (Liu & Spelke, 2017). But it also shows that infants have a model of an objective environment that imposes the same constraints on all agents.

Humans are not the only animals which experience their environment in this way. In the example shown above, the grey ball takes a new path when the barrier is removed. This is an example of a detour problem. More typically this problem arises when the usual path to a goal is blocked, and an alternative route must be found. The ability to solve detour problems is a key piece of evidence that an animal is using a cognitive map for navigation (Tolman, 1948). While it can be challenging to learn to solve a detour problem from scratch, many animals can quickly learn to solve it by watching others (Kabadayi et al., 2018). Dogs and goats, for example, learn quickly from observing humans, and even the solitary red-footed tortoise can quickly solve a detour problem by observing more experienced peers (Wilkinson et al., 2010). These observations suggest, as Hume supposed, that many animals, in addition to humans, represent their environment as objective.

## THE PRESENCE OF OTHERS ENHANCES THE EXPERIENCE OF OBJECTIVITY

In Lau’s example, discussed above, the discriminator was internal, part of the infant’s brain. I suggest that it would often be the child’s carer who confirmed the child’s perception as a true reflection of the world. Likewise, when GANs learn to generate realistic faces, they also need an externally supplied corpus of certified real faces so that the prediction errors, on which learning depends, can be generated. The use of this corpus of real faces is analogous to the carer telling the child what is real. Indeed, the interaction between the generator and the discriminator in a GAN has been described as a form of social learning (Castelle, 2020).

There are, however, mechanisms for enhancing the accuracy and precision of my model of the world which do not require deliberate communication from others. We simply need to observe others. By observing others moving through the environment my feeling of objectivity can be enhanced. A good model enables me to predict my experiences as I move, but a good model also enables me to predict how others will move. They will avoid obstacles. They will move carefully over rough terrain. Successful predictions of the movements of other agents confirm the accuracy of my model of the environment and increase my estimate of its precision, thus increasing the feeling of objectivity. If the movements of others are not constrained in the way I expect, new features of the environment might be revealed. For example, there may be obstacles of which I was unaware. In this way, even observing the movements of rocks rolling down a hill can help me improve the accuracy and precision of my model of the environment.

## PERCEIVING THE WORLD TOGETHER

Unlike rocks, however, humans and many other animals construct internal models of their environment. So, just as I will improve my model of the world by observing others, these others will improve their models by observing me. The various models will become more accurate and precise. In addition, because we are all modelling the same reality, as the models become more accurate, they will also become more similar. This convergence of models will occur automatically. I do not need to know about the other models or even that there are other models. I don’t have to represent your model or your model of my model. As Friston and Frith (2015) have put it “… the infinite regress induced by modelling another agent—who is modelling you—can be finessed if you both possess the same model”. In the case I am considering here, I possess the same model as others, because we are all modelling the same thing, our physical environment.

This internal model of our spatial environment is something we must acquire through experience. In infancy this happens as we move around, interacting with objects by touching, lifting, or dropping, them. Later we might learn from explicit instruction and tools like maps. However, the most effective way to acquire knowledge is through interaction with others. For instance, children can learn a route through a novel environment more effectively by walking with an adult, than by seeing by seeing pictures or a video (Cornell & Hay, 1984). In both humans and other animals, play often involves exploring the environment together, which helps children to develop mapping skills (Blaut & Stea, 1974). In fact, play might well be the primary way by which children learn about their environment (Matthews, 1992).

We observe such engagement in many animals, especially young ones, when play is combined with exploring a new environment. Through these shared experiences, they are developing a non-reflective conscious representation of their environment which is automatically aligned with that of others. This enables them to navigate through a common perceptual space. Deroy and colleagues refer to this as co-perception (Deroy et al., 2024), suggesting we possess a critical ability to distinguish between objects that are perceived by others and those that remain private. The feeling that we are sharing the perception of an object can even impact our sensory abilities. Remarkably, there is an increase in sensitivity (*d*′) for detecting the presence of an object when observers believe that someone else is looking at the same object (Seow & Fleming, 2019). It is easier to perceive the world when we are together with others rather than alone. We have more confidence in what we are seeing.

## THE ADVANTAGES OF COMMON KNOWLEDGE

When we all have the same model of the world, the model is effectively shared between us, even though this sharing is not deliberate. The model we all have has become an implicit form of common knowledge. This confers important advantages. First, as discussed above, a great deal can be learned about the world by simply observing others. Second, holding a model of the world in common greatly enhances the ability to coordinate behaviour. Much has been written about the evolutionary advantages of cooperation for the individual and the group (e.g., Tomasello, 2009). I suggest that, before we achieve cooperation, we need to have coordination. Any kind of joint action needs all the people involved to be coordinated in space. This can be achieved without any additional cognitive load, if everyone has the same internal model of the space they are working in.

## BEYOND THE EXPERIENCE OF SPACE

The mutual knowledge that we share about our spatial environment is like a map. Maps have become important metaphors in neuroscience. The first neural map to be discovered was of the spatial environment (O’Keefe, 1976) and the neural basis of this map is now understood at a computational level in terms of the interaction between place cells (in the hippocampus) and grid cells (in the entorhinal cortex) (Moser et al., 2008). This neural mapping process has now also been found in domains other than space. For example, maps for navigating in social space which take account of identity, sex, hierarchy and affiliation, have been identified in the hippocampus of the fruit bat (Ray et al., 2025). Colours, faces, and even more abstract entities, such as concepts, are also represented as maps (Churchland & Churchland, 2002). Behrens and his colleagues have observed grid cell-like activity in prefrontal cortex when people navigated through a two-dimensional conceptual space (Constantinescu et al., 2016). The suggestion is that these representations may be examples of a general neural mechanism capable of organising all kinds of conceptual knowledge in the form of cognitive maps (Behrens et al., 2018). We might call this a mental landscape.

But do these more conceptual maps also become part of our shared knowledge? We might expect to find examples in maps that are critical for social engagement. Perhaps our most often experienced form of engagement is a conversation. Here successful communication hinges on mutual knowledge (Clark & Marshall, 1981). This shared knowledge allows participants to navigate together through a shared semantic space. Here again this is an objective space in the sense that it is view-point independent and, if communication is to be successful, this semantic space should be sufficiently similar for all of us. Here again we need not be aware of the common knowledge problem. We simply need to know what words mean. We inhabit a common semantic space that feels stable and objective, even if it is so only approximately.

## IMPLICATIONS

In this paper, I have argued that there is a crucial component of sharing in non-reflective conscious experience. This raises an intriguing question: To what extent is this aspect of consciousness dependent on the presence of other people? What happens if we have no one to share with? People kept in solitary confinement, for instance, do not lose consciousness, but their models of the world will no longer be constrained by interactions with others. They will increasingly deviate from reality. This lack of interaction, may also diminish their feeling of objectivity, leading to experiences captured in the terms derealisation and depersonalisation (Gallagher, 2014). Similar experiences are also reported by people with psychosis (Ackner, 1954). This disorder is often characterised as a state in which contact with reality has been lost. However, it might be more accurately described as a loss of contact with the reality normally shared with others. The patient’s beliefs are no longer constrained by interactions with other people (Frith, 2023). They are effectively socially isolated.

Does the idea of sharing offer insights into artificial consciousness? A major reason for thinking that some species other than humans are also conscious is the similarity of their nervous system to ours. But this argument does not work for artificial agents. We need to consider their behaviour. Agents that use large language models (LLMs), such as ChatGPT, are particularly interesting cases because they can engage with us in conversations. Through training on a vast corpus of text these agents have acquired a highly sophisticated representation of semantic space. And, because the LLM has been trained on data from humans, we might plausibly assume that it has acquired the common semantic knowledge that we all share. As a result, we can engage with it productively. But does it understand what we are communicating about? Is it conscious? This topic has sparked intense debate with widely varying opinions (Colombatto & Fleming, 2024). But, even if LLMs don’t really ‘get’ human semantics, when interacting with them people often find the exchange useful and realistic.

There is, however, a key difference between LLMs and humans in relation to the common semantic space that both can create. Humans, unlike LLMs, each have their own (egocentric) point of view of the (allocentric) semantic space they share with others, just as they do with physical space. Their individual point of view derives from their personal experience and their cultural background. Just as we learn about our physical environment by exploring with others, our semantic space emerges from our interactions with family, teachers, and friends and foes. The semantic space acquired by LLMs has no such colour and history and has no such individual perspective. Rather, they exhibit points of view which change depending on the context. LLMs can be seen as having a superposition of a wide range of possible points of view (Kovač et al., 2023). They do not have their own point of view.

## CONSCIOUSNESS—KNOWING TOGETHER

In the not-too-distant past, the word consciousness always implied sharing. It derives from the Latin *conscio*, ‘I know together, I have joint or common knowledge with another’. For example. Hobbes wrote, ‘*When two, or more men, know of one and the same fact, they are said to be CONSCIOUS of it one to another; which is as much as to know it together*’ (Hobbes, 1651/1996, 7, 4). And this usage of the term *consciousness* was still to be found in the writings of English authors up to the 19^th^ century. In Jane Austin’s *Northanger Abbey* (1818), Henry Tilney is introduced to Mrs. Moreland ‘*by her conscious daughter*’. Meaning that Catherine, the daughter, has secret, shared knowledge with Henry, knowledge that is not known to her mother (Lewis, 1960, p. 186).

In this paper, I suggest that we should not forget this social aspect of the word consciousness. The idea that consciousness (regardless of whether it is reflective or non-reflective) has a built-in sharing component highlights two remarkable aspects of our experience. First, there is the merging of the subjective with the objective. Our experience of space is subjective in the sense that it is constructed by our private sensations and expectations, and yet we experience it as objective. Second, there is the merging of the individual and the group. As a group, we have a common knowledge of space, but we also remain aware of our individual viewpoint. It is these combinations that enable us to coordinate with each other within a shared conscious space without needing to reflect on our subjective experience or to intentionally communicate its content to others. This automatic form of coordination provides many advantages for group interactions and is a necessary precursor to the evolution of cooperation.

## ACKNOWLEDGMENTS

CDF is not in receipt of any funding but is grateful for support from UCL and the University of London. I thank Nick Shea, Matan Mazor, Hakwan Lau, Bahador Bahrami, Stephen Fleming, Rosalind Ridley, Uta Frith, and two anonymous reviewers for their most helpful comments on earlier drafts of this paper.

## Note

^1^ I sometimes lose this accuracy of prediction for a while when getting used to a new pair of glasses.
